# A Woman's Experience in a General Hospital
*The patient whose views are expressed in this article was an in-patient in a large general hospital. where she was operated on for carcinoma of the breast. The additions in brackets are explanatory of statements made in the text.

**Published:** 1913-07-19

**Authors:** 


### HOSPITALS FROM THE PATIENTS' POINT OF VIEW.

(Contributions to this Section are Invited.)

### A Woman's Experience in a General Hospital.*

I was sent in by my own doctor, who told me that an operation was necessary, and that it would be a severe one, but the only means left to me, as I had a cancer which could not be otherwise cured. It was explained to me that the operation held out some hope of recovery from the disease; but before I entered the hospital I did not know what exactly was the nature of the operation, nor was I aware that there were students and that I would have to be shown to them. I travelled down and came to the hospital on receipt of instructions from my doctor. I was told to take with me certain articles of underwear and personal necessities, such as a dressing gown, bed socks, brush and comb, tooth brush, and so on. But I must say that I omitted several things in my hurry, and it seems to me that if the hospital authorities would have a printed paper which could give patients like myself some hints as to what to bring, it would be highly appreciated. Some of us, I feel sure, do not like to trespass on the kindness of the nurses and the hospital in asking for comforts which we might easily have brought with us from home. Perhaps the hospital prefers to have everything of one pattern, and there are many patients who cannot afford such comforts and who must therefore depend on the institution; but those who can afford them could easily bring them if they only knew what would be required and what would not be tolerated.

I was placed in a fairly large ward, occupied by women and children. Between the bed I occupied and the bed on my right was a cot in which was a nice little baby, who had its leg fastened up to a kind of wheel above the bed. [This was a case of fractured thigh.] On my left was a bed occupied by an old woman. She was a dear old lady, and never complained, while she apologised to all of us, for her condition made it necessary that she should be dressed several times a day. This dressing was the first unpleasant thing that I became aware of. Screens were placed round the bed, and none of us could see what occurred; but there was always a most unpleasant smell which lingered for a long time although the ward was well aired. [The case was one of intestinal obstruction in which colotomy had been performed: the smell was due to the removal of the dressings from the colotomy wound when impregnated with fæces.] It struck us all at the time that something might have been done to avoid that unpleasantness [*i.e.* by dressing the case outside the ward when necessary]. Later on, after I had been operated on, I must say I did not like the baby nearly so much as before; the poor thing sometimes cried very bitterly, and none of us were able to comfort it, while the nurses were too busy sometimes to cheer it up.

The first evening in the ward I could hardly sleep, owing to the excitement and the strangeness of my surroundings; but I dozed off in the early morning, only to be awakened by the ward maids, who came in with a clatter to scrub the floor and clean up things. It seemed to me that they started much too early, but the nurses told me this had to be done else they would never get through the day's work. We got our breakfast very early too. I had tea and bread and butter and an egg, but the tea was very bitter and I could scarcely swallow it. I had made up my mind that I would not complain, for I felt sure that everyone was doing his utmost for me, and they were all so kind; but I must say that I often found the food given out stale and not at all like what I had been used to at home. When the house surgeon came round he was very kind and asked me if I was comfortable and wanted anything else. Afterwards the other surgeon came—I think he was one of the students—and asked me questions and examined me. I did not think that would have to be done and rather dreaded it at first, but he and the nurse who was with him were very kind and put screens round the bed, so that the examination was quite private. In the afternoon the head surgeon and some students came round, and I was again examined, while the head surgeon explained my case to them. I had dreaded this too; but it was really not so unpleasant as I fancied it might be.

On the second day nurse came and prepared me for the operation, which I was told would take place in the afternoon of the third day. By this time I had talked to some of the other patients, one of whom had to be operated on the same day; and I had got over my shyness and become used to the ward as it were. We were not disturbed by noise—except from the baby—and I slept quite well. I did not like the dressing they put on my chest at first it was so clammy and wet; but I got used to it and slept all right. The next morning I had a very light breakfast, and nothing except a cup of tea at midday. I felt very hungry, but I was told that I could not have anything more else I would not be able to take the chloroform. The operation was to be at two o'clock that afternoon, and I lay waiting ever so long a time, wondering about it, until sister came and told me they could not do it that afternoon and asked nurse to give me something to eat. [This postponement of a major operation, with the concomitant psychical distress it occasions the patient, cannot always be avoided; but its effects may be minimised by proper management: in this case the patient might have been allowed a light meal in the early afternoon if the rota of operation cases made it impossible to proceed with her case until four o'clock; at two o'clock it would have been possible to have notified her that her case could not be taken owing to press of work.]

I was operated upon three days later. The only

* The patient whose views are expressed in this article was an in-patient in a large general hospital, where she was operated on for carcinoma of the breast. The additions in brackets are explanatory of statements made in the text.

thing I remember is that the chloroform [as a matter of fact A.C.E. mixture was used] made me feel very sick afterwards, and I did not mind the pain nearly as much as the sickness. They gave me something for it but it did not help me, and I retched a good deal for almost a day and felt very uncomfortable. I had an injection that night and slept by fits and starts; but the ward maids always woke me early in the morning, and I thought them a great nuisance. The nurses were very kind; they made the bed without giving me any discomfort, although I had to move a bit while they did so; and it seemed to me at first that my arm, which was bound to my chest, would pain me. One thing I recollect troubled me much more than that—the light from the window, which always fell directly on my face; there was no blind, and though at first the screens sheltered me, when they were taken away I had to look directly at the window opposite. [The interposition of a bed-screen would have obviated this.] Later on I was continually shifting down in the bed; they propped me up with pillows, but that did not seem to suit; the pillows were always shifting, and I sometimes felt that things would have been better if they had placed my locker behind me for me to lean against. [A solid bed-rest was wanted here; obviously the adjustable back-rest attached to the bedstead did not act well.]

When I was better and could read I found that I was not allowed to do so in the evening; all the lights were turned down, and I could not have a bed light as they said it was against the rules. So I had to lie awake for hours until the house surgeon came round late in the night; after that I dropped off, though I sometimes had a draught before I could go to sleep. As far as the nursing was concerned I was excellently treated, and had nothing whatever to complain about.

My fellow-patients were of all sorts, but they were all sympathetic and kind. Number 24 was a lady who came in a few days before me and had a very serious operation; the screens were always around her bed, and we knew that she was doing badly. Then one morning we somehow heard that she was dying. This was the most unpleasant time I had, for we all felt that Number 24 was very seriously ill, and most of us could see nurses and doctors going and coming from her bed. We could hardly talk as it did not seem quite the thing to do. The worst of it all was when two porters came in with a bier and carried her away. I thought they might have done things a bit more decently and kept it from us. [The subject of the treatment of a dying patient in a general ward is one that needs serious consideration. From this patient's statement it is evident that the removal of Number 24 to a private ward or "dying room" even would not have caused the psychical distress that the removal of the body, with the inseparable trappings used in hospital, occasioned.]

